# No Xenotropic Murine Leukemia Virus–related Virus Detected in Fibromyalgia Patients

**DOI:** 10.3201/eid1702.100978

**Published:** 2011-02

**Authors:** Joanna Luczkowiak, Olalla Sierra, Jorge Juan González-Martín, Gabriel Herrero-Beaumont, Rafael Delgado

**Affiliations:** Author affiliations: Hospital Universitario 12 de Octubre, Madrid, Spain (J. Luczkowiak, O. Sierra, R. Delgado);; IIS-Fundación Jiménez Díaz, Madrid (J.J. González-Martín, G. Herrero-Beaumont)

**Keywords:** Viruses, xenotropic murine leukemia virus, XMRV, fibromyalgia, letter

**To the Editor:** Xenotropic murine leukemia virus–related virus (XMRV) is a recently described human retrovirus that has been associated with prostate cancer and chronic fatigue syndrome (CFS) (*1**,**2*). XMRV is similar to a classic murine endogenous leukemia retrovirus, murine leukemia virus (MLV), which infects strains of mice that do not express the specific viral receptor. XMRV is genetically close to, although differentiable from, MLV. The first evidence of its presence in humans was obtained by Urisman et al. in prostate cancer tissue (*1*). In 2009, Lombardi et al. (*2*) found XMRV sequences and specific antibody responses in 67% of a large group of patients with CFS in North America. This association was notable because XMRV sequences were found in only 4% of healthy controls. These results have generated controversy because several independent studies, mainly in Europe (*3**–**5*) but also in North America (*6*), have been unable to detect XMRV sequences in patients with CFS. Furthermore, a recent report from North America (*7*) appears to confirm the initial results by Lombardi et al. (*2*) in patients with CFS and expands the viral association to a wider variety of XMLV-related viruses that seem closer to polytropic mouse endogenous retroviruses.

Fibromyalgia is a multifactor condition characterized by widespread pain and diffuse tenderness. Although trauma and stress can worsen or even precipitate development of the syndrome, infections with certain viruses, including hepatitis C virus and HIV, have been associated with development of fibromyalgia (*8*). Nevertheless, fibromyalgia remains a disease of unknown etiology. Although CFS is a distinct entity, features shared by both diseases suggest that CFS and fibromyalgia represent the same underlying condition (*9*). Additionally, because they are often accompanied by a noticeable mental health effect (*9*), the presence of a potential neurotropic retroviral agent in both diseases could explain these similarities. Therefore, we studied the presence of XMRV and polytropic MLV–related retroviruses in a group of patients with fibromyalgia.

During January 2010, blood samples were collected from 15 patients in whom fibromyalgia had been previously diagnosed according to American College of Rheumatology criteria (www.rheumatology.org/practice/clinical/classification/fibromyalgia/1990_Criteria_for_Classification_Fibro.pdf). Ten healthy blood donors served as controls. For XMRV screening, we used DNA extracted from 400 μL of whole blood collected in EDTA tubes by the QIAamp DNA Mini Kit (QIAGEN, Hilden, Germany). Nested PCR was done by using 5 sets of primers corresponding to the *gag* (*3*) and *env* (*2*) regions of XMRV as described (*2**,**3*,*7*). The first round of PCR was conducted by using 500 ng of genomic DNA, equivalent to 7.5 × 10^4^ nucleated blood cells, in a final volume of 50 μL, by using the Expand High Fidelity PCR System (Roche Applied Science, Basel, Switzerland). A second round of PCR was conducted under the same conditions by using 5 μL of the first reaction product. Details of the nested-PCR strategy were as follows: *gag* region was amplified by outer primers 419F and 1154R (*2*) and 3 sets of inner primers: XMRV-FI-441/XMRV-RI-566 (*3*), MLV-GAG-I-F/MLV-GAG-I-R, and MLV-NP116/MLV-NP117 (*7*). Nested PCR for *env* was performed by using outer primers 5922F and 6273R (*2*) and 2 sets of inner primers: 5922F/6173R and 5942F/6159R (*7*). Primers for human β-globin were used as positive controls of human DNA amplification (*3*). The full-length molecular viral clone VP62 (obtained through the National Institutes of Health AIDS Research and Reference Reagent Program [Rockville, MD, USA] from R.H. Silverman and B. Dong) (*10*) was used as a positive XMRV control. All samples were examined on a 2% agarose gel stained with ethidium bromide (Figure). The overall sensitivity of the nested PCR procedure, estimated by spiking VP62 into negative samples, was 1–10 copies per sample.

Using highly sensitive PCR tools and a multiple set of primers to detect xenotropic and polytropic MLV–related sequences, we found no evidence of MLV-related sequences in blood cells from fibromyalgia patients or controls. Our results agree with those from studies of CFS cohorts in Europe and North America that also failed to confirm XMRV in blood samples (*3**–**6*). Technical issues or geographic specificities probably could not account for such a difference; therefore, these negative results raise concerns about the role of XMRV in these syndromes. Nevertheless, with this relatively small population we cannot absolutely exclude an association of XMRV or polytropic MLV–related viruses with fibromyalgia. However, a proportion of fibromyalgia cases with XMRV >22% would be unlikely (3/15 cases, 95% confidence interval 0–3), which is clearly insufficient to support a significant association between XMRV and fibromyalgia.

Fibromyalgia does not appear to be associated with XMRV or polytropic MLV–related viruses. The role of these new agents in human disease, and specifically in CFS, remains to be clearly confirmed in multicenter and standardized studies.

**Figure:**
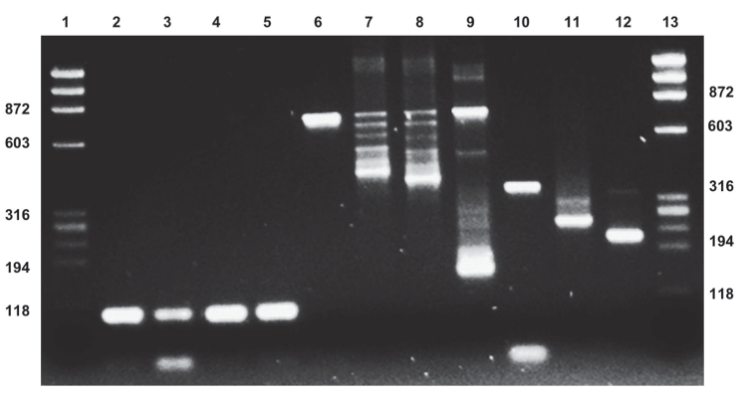
Testing for xenotropic murine leukemia virus–related virus (XMRV) in patients with fibromyalgia. Lanes 1 and 13, molecular weight marker ΦX174RF *Hae*III; lanes 2–5, hBG for patients 1–4 (primers: hBG-FI-170/hBG-RI-273 (103 bp); lanes 6–12, positive control (pcDNA3.1-XMRV-Vp62) 1,000 copies (lanes 6 and 10) and 100 copies (lanes 7–9 and 11–12); lane 6, primers *gag* 419F/1154R (735 bp); lane 7, primers *gag* MLV-GAG-I-F/MLV-GAG-I-R (413 bp); lane 8, primers *gag* MLV-NP116/MLV-NP117 (380 bp); lane 9, primers *gag* XMRV-FI-441/XMRV-RI-566 (125 bp); lane 10, primers *env* 5922F/6273R (351 bp); lane 11, primers *env* 5922F/6173R (252 bp); lane 12, primers *env* 5942F/6159R (218 bp).
